# Selection Signature and CRISPR/Cas9-Mediated Gene Knockout Analyses Reveal *ZC3H10* Involved in Cold Adaptation in Chinese Native Cattle

**DOI:** 10.3390/genes13101910

**Published:** 2022-10-20

**Authors:** Luyu Wang, Yaping Gao, Jinpeng Wang, Ning Huang, Qiang Jiang, Zhihua Ju, Chunhong Yang, Xiaochao Wei, Yao Xiao, Yaran Zhang, Ling Yang, Jinming Huang

**Affiliations:** 1 Key Laboratory of Livestock and Poultry Multi-Omics of MARA, Institute of Animal Science and Veterinary Medicine, Shandong Academy of Agricultural Sciences, Jinan 250100, China; 2 Shandong Key Laboratory of Animal Disease Control, Breeding and Reproduction, Jinan 250100, China; 3 Department of Animal Science, School of Life Science and Food Engineering, Hebei University of Engineering, Handan 056038, China

**Keywords:** *ZC3H10*, cold adaptation, transcriptome, CRISPR/Cas9, cattle

## Abstract

Cold stress is an important factor affecting cattle health, production performance, and reproductive efficiency. Understanding of the potential mechanism underlying genetic adaptation to local environments, particularly extreme cold environment, is limited. Here, by using FLK and hapFLK methods, we found that the Zinc finger CCCH-type containing 10 (*ZC3H10*) gene underwent positive selection in the Menggu, Fuzhou, Anxi, and Shigatse humped cattle breeds that are distributed in the cold areas of China. Furthermore, *ZC3H10* expression significantly increased in bovine fetal fibroblast (BFF) cells at 28 °C for 4 h. ZC3H10 knockout BFFs were generated using CRISPR/Cas9. Wild and ZC3H10-deleted BFFs were treated at two temperatures and were divided into four groups (WT, wild and cultured at 38 °C; KO, ZC3H10^−/−^ and 38 °C; WT_LT, wild, and 28 °C for 4 h; and KO_LT, ZC3H10^−/−^ and 28 °C for 4 h. A total of 466, 598, 519, and 650 differently expressed genes (two-fold or more than two-fold changes) were identified by determining transcriptomic difference (KO_LT vs. KO, WT_LT vs. WT, KO vs. WT, and KO_LT vs. WT_LT, respectively). Loss of ZC3H10 dysregulated pathways involved in thermogenesis and immunity, and ZC3H10 participated in immunity-related pathways induced by cold stress and regulated genes involved in glucose and lipid metabolism and lipid transport (PLTP and APOA1), thereby facilitating adaptability to cold stress. Our findings provide a foundation for further studies on the function of ZC3H10 in cold stress and development of bovine breeding strategies for combatting the influences of cold climate.

## 1. Background

After long-term natural and artificial selection and domestication, many local cattle breeds have adapted well to the challenges of local environments, including extremely low temperatures. Cold climate is one of the most important external environmental factors that affect cattle health, production performance, and reproductive efficiency [1]. Understanding the genetic basis of cold adaptation is very helpful to the design of effective breeding strategies for protecting local breeds and improving commercial cattle.

China’s climate is complex and diverse. In winter, the temperature difference between north and south in China is large; the south is warm, and the north is cold. Therefore, these well-adapted cattle breeds are distributed in north China and the Qinghai-Tibet Plateau and are good models for uncovering the molecular basis of cold adaptation. Selection signature analysis aids the identification of genes related to phenotypic traits [2]. Many approaches can be used, including integrated haplotype score [3], cross-population-extended haplotype homozygosity [4], hapFLK, and FLK [5]. The common strategy for detecting selection signatures is to compare two distinct populations and then searching genomic regions with outstanding genetic differentiation. Chinese native cattle breeds exhibit a complex admixture of components and history of breed formation [6]. The hapFLK test was used in identifying selective regions and genes involved in cold adaptation because of its robust performance when resolving hierarchical structures [5].

Zinc finger CCCH-type containing 10 (ZC3H10) is a critical regulator of the early stages of adipogenesis [7]. The depletion of *Zc3h10* in mouse preadipocytes results in reduced lipid accumulation, whereas the overexpression of *Zc3h10* increases lipid droplet size and reduces the number of mature adipocytes [8]. ZC3H10, a mitochondrial regulator, has an important impact on growth and activity of mitochondria which produce ATP to supply energy [9]. However, the role of *ZC3H10* in cattle has not been elucidated yet.

We hypothesized that the *ZC3H10* gene is subjected to strong positive selection in cold-adapted cattle breeds. Here, we used our previously reported 777K SNP genotyping data of Chinese breeds to identify the genomic signatures of adaptation to cold climate and determine whether *ZC3H10* has significant positive selective signals in northern Chinese and high-altitude cattle breeds. Furthermore, we constructed a bovine *ZC3H10* knockout cell model with the CRISPR/Cas9 technology and explored cold-stress-induced changes in the transcriptome of *ZC3H10*^−/−^ fetal bovine fibroblasts to study the possible role of *ZC3H10* in bovine cold adaptation.

## 2. Material and methods

### 2.1. FLK and hapFLK Genomic Scans and Local Tree Building of ZC3H10

**Figure 1:**
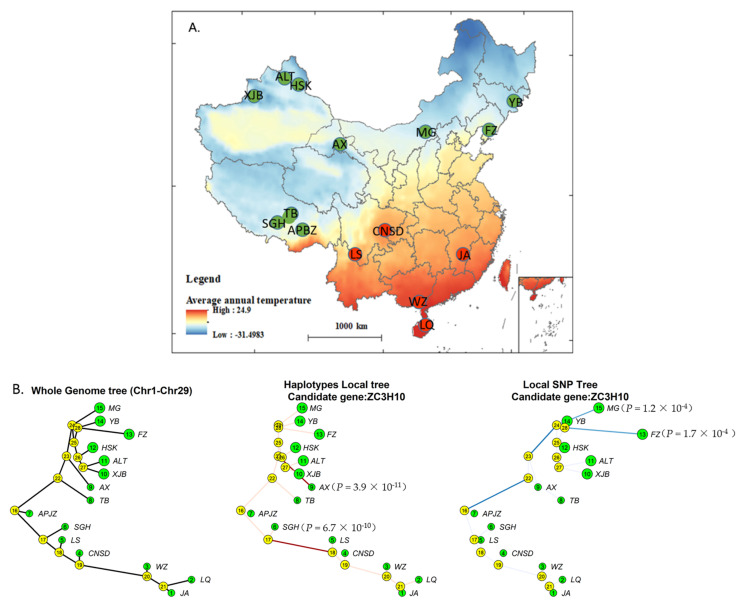
Distribution and selective signature of 15 cattle breeds. (**A**) Distribution of sampling cattle breed. Green circles represent the cold-adapted group, red circles indicate the heat-adapted group. (**B**) Local trees of the bovine *ZC3H10* from the FLK and hapFLK analyses. The *p* value represents the significance of selection signatures.

A 777K SNP genotyping dataset including 15 Chinese indigenous cattle breeds and Nelore cattle (Table S1; Figure 1A) was used in running hapFLK programs for selection signature analysis [5]. The dataset was extracted from our previous publication [6]. Cattle breeds distributed in areas with an annual average temperature lower than 10.5 °C were classified as the cold-adapted group, whereas those distributed in an area with an annual average temperature higher than 18 °C were considered the heat-adapted group (Figure 1A). Nelore cattle were used as the outgroup. The signatures of selection between the cold-adapted and heat-adapted groups were compared using the FLK and hapFLk statistics. Two revised scripts (local_reynolds.py and local_trees. R) were used in building and plotting local population trees of the candidate *ZC3H10* gene for the identification of selected populations.

### 2.2. RT-PCR

Total RNA was extracted from the heart, liver, spleen, lungs, and kidney of cattle using TRIzol reagent (15596018, Thermofisher, Carlsbad, CA, USA) according to the manufacturer’s procedure. cDNA was prepared using a FastKing RT kit (KR116-02, TIANGEN, Beijing, China). The relative expression of *ZC3H10* in different tissues of cattle using 2 × UrTaq Master Mix (R232, Nobelab) were determined by RT-PCR according to the manufacturer’s instructions. The bovine *ZC3H10* primers for RT-PCR were 5′-CGGACTTCACCTGAGCTACC-3′ and 5′-CTGTCCCACTGGCCTCCT-3′ (size = 167 bp).

### 2.3. Construction and Transfection of ZC3H10 Gene Knockout Vector

A guide RNA sequence (gRNA: 5′-CACCgcatggatgaatcggcagtta-3′) for CRISPR/Cas9 was designed to target the exon 3 of the bovine *ZC3H10* gene (GenBank AC_000162.1). The complementary oligonucleotides for guide RNA (gRNA) were annealed and cloned into the pGK1.1 CRISPR/Cas9-Puro vector (Genloci Biotech, Nanjing, China). Bovine fetal fibroblasts (BFFs) were transfected with pGK1.1/gRNA using lipofectamine 3000 (L3000001, Invitrogen, Carlsbad, CA, USA) according to the manufacturer’s instructions. Two days after transfection, the cells were treated with 2.5 μg/mL puromycin for 3 days. After 2 weeks, colonies were isolated with cloning cylinders, and *ZC3H10* sequences were analyzed through DNA sequencing and RT-PCR. The primers for DNA sequencing were 5′-GGCAGAGTGCTCATCCGTTAG-3′ and 5′-TCATTGGTAGCCAGAAGGT-3′ (size = 999 bp). Primers for RT-PCR were 5′-AACTGCCGATTCATCCATGG-3′ and 5′-AAAATCCCGTTGCAGGTGAC-3′ (size = 219 bp).

### 2.4. Cell Culture and Cold-Stress Treatment

BFFs were cultured in DMEM-high glucose (C11995500BT, Gibco, Grand Island, New York, USA) containing 10% FBS (10099141, Gibco) in a 5% CO_2_ atmosphere at 38 °C. The temperature of the cell incubator was adjusted to 28 °C for 4 h. The cells were divided into four groups: WT (wild and 38 °C), KO (*ZC3H10*^−/−^ and 38 °C), WT_LT (wild and treated at 28 °C for 4 h), and KO_LT (*ZC3H10*^−/−^ and treated at 28 °C for 4 h).

### 2.5. Library Construction and Sequencing

Total RNA was extracted from the four groups with TRIzol reagent (15596018, Thermofisher) according to the manufacturer’s procedure. The quantity and purity of total RNA were analyzed with Bioanalyzer 2100 and RNA 6000 Nano LabChip Kit (5067-1511, Agilent, Beijing, China). High-quality RNA samples with RIN number of >7.0 were used in constructing sequencing libraries. After total RNA was extracted, mRNA was purified from total RNA (1 µg) with Dynabeads Oligo (dT) (Thermo Fisher), and two rounds of purification were performed. After purification, mRNA was fragmented into short fragments by using divalent cations under elevated temperature (magnesium RNA fragmentation module [e6150, NEB] under 94 °C 5–7 min). Then, the cleaved RNA fragments were reverse-transcribed for the preparation of cDNA with SuperScript II reverse transcriptase (1896649, Invitrogen). The cDNA was used in synthesizing U-labeled second-stranded DNAs with *Escherichia coli* DNA polymerase I (m0209, NEB), RNase H (m0297, NEB), and dUTP Solution (R0133, Thermo Fisher). An A-base was then added to the blunt ends of each strand, which was prepared for ligation to indexed adapters. Each adapter contained a T-base overhang for ligating an adapter to an A-tailed fragmented DNA. Dual-index adapters were ligated to fragments, and size selection was performed with AMPureXP beads. After the heat-labile UDG enzyme (m0280, NEB) treatment of the U-labeled second-stranded DNAs, the ligated products were amplified with PCR under the following conditions: initial denaturation at 95 °C for 3 min; eight cycles of denaturation at 98 °C for 15 s, annealing at 60 °C for 15 s, and extension at 72 °C for 30 s, and then final extension at 72 °C for 5 min. The average insert size for the final cDNA libraries was 300 ± 50 bp. Finally, 150 bp paired-end sequencing (PE150) was performed twice on an Illumina Novaseq 6000 (LC-Bio Technology CO., Hangzhou, China) according to the vendor’s recommended protocol.

### 2.6. Reads Filtering and Differential Expression Analysis

By using the Illumina paired-end RNA-seq approach, the transcriptome was sequenced. A total of million 2 × 150 bp paired-end reads was obtained. In accordance with the requirement of bioinformatics analysis, clean reads were obtained by removing contaminated reads (reads containing adapters or poly-N and low-quality reads) from the raw reads by using Cutadapt (https://cutadapt.readthedocs.io/en/stable/, accessed on 20 November 2021; version: cutadapt-1.9). Then, Q20, Q30, and GC content of the clean data were calculated. All the downstream analyses were based on clean data of high quality. Clean reads were mapped to the cow reference genome by using the HISAT2 package (https://daehwankimlab.github.io/hisat2/, accessed on 2 December 2021; version: hisat2-2.0.4). The mapped reads of each sample were assembled using StringTie (http://ccb.jhu.edu/software/stringtie/, accessed on 15 December 2021; version: stringtie-1.3.4d) with default parameters. Then, all transcriptomes from all samples were merged for the reconstruction of a comprehensive transcriptome with gffcompare software (http://ccb.jhu.edu/software/stringtie/gffcompare.shtml, accessed on 22 December 2021; version: gffcompare-0.9.8). After the final transcriptome was generated, StringTie and ballgown (http://www.bioconductor.org/packages/release/bioc/html/ballgown.html, accessed on 25 December 2021) were used in estimating the expression levels of all transcripts and the expression abundance for mRNAs by calculating the fragment per kilobase of transcript per million mapped reads (FPKM). Analyses of the differentially expressed genes (DEGs) were performed with DESeq2 software between two different groups (and by edgeR between two samples). The genes with a parameter of false discovery rate (FDR) below 0.05 and absolute fold change of ≥2 were considered DEGs.

### 2.7. Enrichment Analysis of GO and KEGG

The DEGs of four comparisons (WT vs. WT_LT, KO vs. KO_LT, WT vs. KO, and WT_LT vs. KO_LT) were identified before Gene Ontology (GO) enrichment analyses (http://geneontology.org, accessed on 27 December 2021) and Kyoto Encyclopedia of Genes and Genomes (KEGG) analyses (https://www.kegg.jp/kegg/, accessed on 27 December 2021). A GO term or pathway with corrected *p* value of ≤0.05 was defined as significantly enriched in DEGs.

### 2.8. Establishment of a PPI Network

STRING (Version: 11.5; https://string-db.org/, accessed on 10 March 2022) online database [10] and Cytoscape (version 3.9.1) software (http://www.cytoscape.org/, accessed on 11 March 2022) were used in visualizing and analyzing the PPI network.

### 2.9. Validation of Gene Expression Using RT-qPCR

Total RNA was extracted from fetal bovine fibroblasts with TRIzol reagent (15596018, Thermofisher) according to the manufacturer’s procedure. cDNA was prepared using a FastKing RT kit (with gDNase; KR116-02, TIANGEN). The relative expression of PLTP and APOA1 mRNA in the BFFs of WT (n = 3), KO (n = 3), WT_LT (n = 3), and KO_LT (n = 3) was determined through RT-qPCR. Specific PCR primers (F:5′-AGGGAATCGACTTTGTGCGT-3′; R:5′-GGGCCGGTTCTTCTCAATCA-3′) were designed to amplify a 113 bp product from PLTP and specific primers (F:5′-TGCTGGCCATTGAGGTCAC-3′; R:5′-TCAGGAAGAGCACAGCCAAG-3′) to amplify a 74 bp product from APOA1 using ChamQ Universal SYBR qPCR master mix (Q711-02, Vazyme, Nanjing, China) according to the manufacturer’s protocol. The 20 μL RT-qPCR mixture contained 10.0 μL of ChamQ Universal SYBR qPCR master mix (Q711-02, Vazyme), 0.5 μM forward and reverse primers, 2.0 μL of cDNA (100 ng), and 7 μL of ddH_2_O. RT-qPCR was performed using a Roche LightCycler 480 system (Roche Applied Science, Mannheim, Germany) under the following conditions: 95 °C for 30 s (denaturing), 40 cycles at 95 °C for 5 s, and 60 °C for 30 s. The last stage for the dissociation curve was as follows: 95 °C for 15 s, 60 °C for 60 s, and 95 °C for 15 s. Then, hold at 50 °C for 30 s (cooling). Target gene expression was calculated relative to the housekeeping gene β-actin (F:5′-CATCGGCAATGAGCGGTTCC-3′; R:5′-ACCGTGTTGGCGTAGAGGTC-3′; size = 147 bp). Each sample was measured in triplicate, and the experiment was repeated at least three times. Data were analyzed using the 2^−ΔΔCt^ method. Data were expressed as mean ± SE values. Differences between any two groups were analyzed using Student’s *t*-tests. A *p* value of less than 0.05 was considered statistically significant.

## 3. Results

### 3.1. Signatures of Selection in the Genomes of Cattle Breed in Cold and Heat Areas

Fifteen Chinese local cattle breeds were divided into cold-adapted and heat- adapted groups on the basis of local annual average temperature. FLK and hapFLK tests were used to compare selection signatures on the autosome genome, and the top 0.1% of SNPs diverged in allele frequencies between two groups (Table S1) were used in characterizing the positive selective regions and genes that were putatively responsible for temperature adaptation. As a result, 187 and 116 genes were detected by FLK, and hapFLK. Seventy-four genes were identified by both of methods, including *ZC3H10*.

Through FLK and hapFLK analyses, the SNP and haplotype local trees of *ZC3H10* were built and used in determining which cattle breed was positively selected. The results showed that *ZC3H10* had undergone significant positive selection in the Menggu (MG; P_FLK_ = 1.2 × 10^−^^4^) and Fuzhou (FZ; P_FLK_ = 1.7 × 10^−4^) cattle breeds in the FLK, and in the Anxi (AX) (P_hapFLK_ = 3.9 × 10^−11^) and Shigatse humped (SGH; P_hapFLK_ = 6.7 × 10^−10^) cattle breeds in the hapFLK (Figure 1B). All the four selected cattle breeds are in cold areas and have good adaptability to cold environments, suggesting that *ZC3H10* is involved in cold adaptation in cattle.

### 3.2. Expression of ZC3H10 mRNA in Bovine Different Tissues and in Cold-Treated Cells

**Figure 2:**
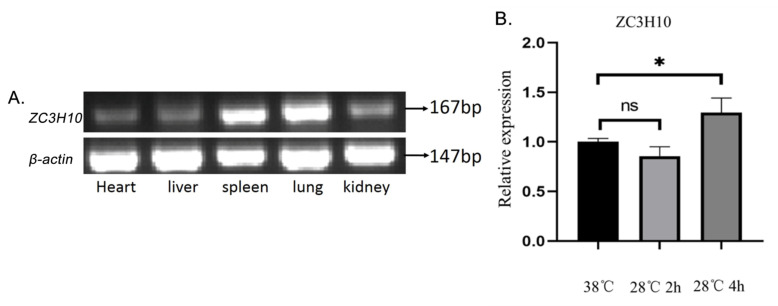
Expression of *ZC3H10* mRNA in different tissues and in BFFs at different temperatures and treatment times. (**A**) Expression of *ZC3H10* mRNA in different tissues. (**B**) Expression of *ZC3H10* mRNA in BFFs at different temperature and treatment times. * *p* < 0.05.

The mRNA expression levels of different tissues were detected by RT-PCR and used in investigating the expression of *ZC3H10*. The results show that *ZC3H10* was expressed in bovine heart, liver, spleen, lungs, and kidneys, and the expression levels in the spleen and lungs were higher than those in other tissues (Figure 2A). Compared with the non-cold-treated BFFs, no significant difference in *ZC3H10* expression was found in cells after treatment at 28 °C for 2 h, and the expression significantly increased in cells after a 28 °C treatment for 4 h (Figure 2B), indicating *ZC3H10* is associated with cold stress. However, the underlying mechanism is unclear.

### 3.3. Production and Screening of ZC3H10 Knockout Cell Line

**Figure 3:**
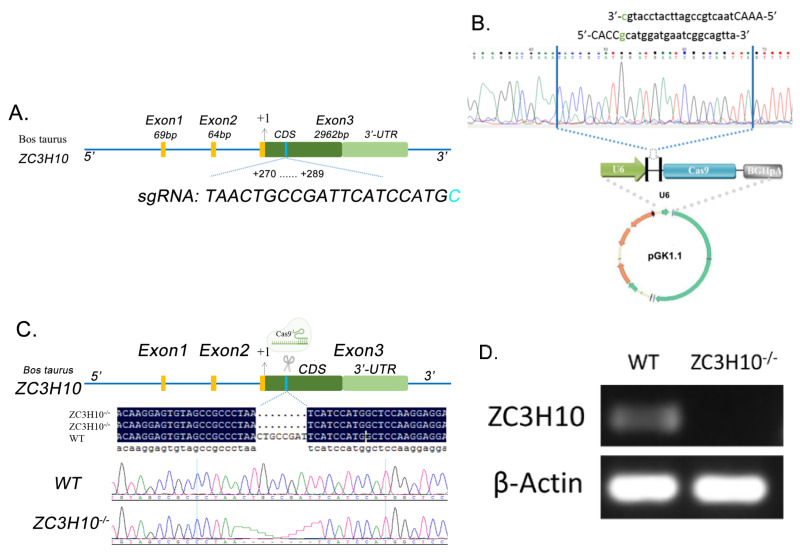
Construction of *ZC3H10*^−/−^ bovine fibroblast cells. (**A**) Structure of *ZC3H10* and design of gRNA sequence of CRISPR/Cas9. (**B**) Construction of the plasmid of pGK1.1-ZC3H20. (**C**) Validation of deletion sequences. (**D**) RT-PCR result of wild and *ZC3H10*^−/−^ BFFs.

To examine the function of *ZC3H10*, we generated a *ZC3H10* knockout fibroblast cell line (*ZC3H10*^−/−^) with a CRISPR/Cas9 technology. According to the reference sequence of bovine *ZC3H10* (GenBank AC_000162.1), candidate gRNAs for CRISPR/Cas9 were designed using CRISPRdirect [11] to target the exon 3 of *ZC3H10*. Online sequence alignment was carried out in combination with Ensembl to ensure its specificity. Finally, gRNA was determined, and the selected location of gRNA was located at 270–289 bp in the CDS region of *ZC3H10* (Figure 3A). The complementary oligonucleotides for gRNA were annealed and inserted into the pGK1.1 vector (Figure 3B). The pGK1.1/gRNA vector was transfected into the BFFs. After puromycin treatment and isolation of colonies, the *ZC3H10*^−/−^ mutant was generated by deleting an 8 bp region of exon 3, which produced a missense segment after the first 91 amino acids and introduced a premature stop codon, which was 99 amino acids away from the lesion site (Figure 3C). The RT-PCR result showed that *ZC3H10* was successfully knocked out (Figure 3D).

### 3.4. Overview of RNA Sequencing Data

**Figure 4:**
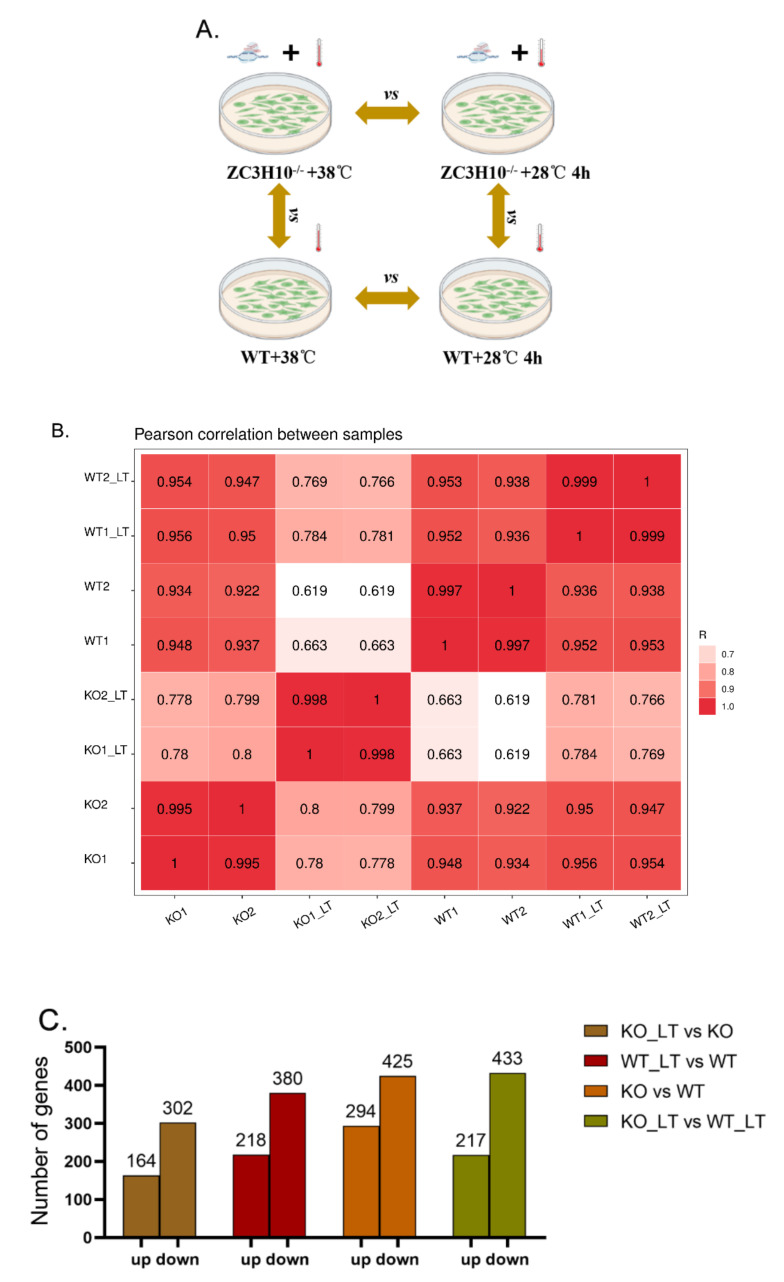
Experimental design, correction of sequencing samples, and number of DEGs in different comparisons. (**A**) Four treatments. (**B**) Person correlation of sequencing samples. (**C**) Up-regulated and down-regulated DEGs in four comparisons.

To evaluate the transcriptomic change of *ZC3H10*^−/−^ BFFs under cold stress, the cells were cultured at 28 °C for 4 h. The RNA-seq of the four groups was performed (Figure 4A). The quality inspection results of each sample met the standards of database establishment and the requirements of subsequent experiments (Table S2). A total of 7.6 G raw bases and 6.49 G valid data bases were obtained. A Q20 of ≥99% and Q30 of ≥98% were obtained (Table S3). The clean reads of the eight samples were assembled and mapped to the bovine genome. Among the eight samples, the percentages of the total mapped and uniquely mapped reads for the eight libraries were greater than 82% and 76%, respectively (Table S4). Pearson correlation coefficients between samples in the same group were all higher than 0.995 (Figure 4B), indicating that the samples have good repeatability. The DEGs in BFFs between WT and WT_LT, KO and KO_LT, WT and KO, and WT_LT and KO_LT were analyzed by setting the following reference values: |log_2_FC| ≥ 1, FKPM > 20, and FDR < 0.05. In total, 466, 598, 519, and 650 DEGs were identified in four comparisons (KO_LT vs. KO, WT_LT vs. WT, KO vs. WT, and KO_LT vs. WT_LT; Figure 4C; Table S5).

### 3.5. ZC3H10 Knockout in BFFs Dysregulated Pathways Associated with Thermogenesis and Immunity

**Figure 5:**
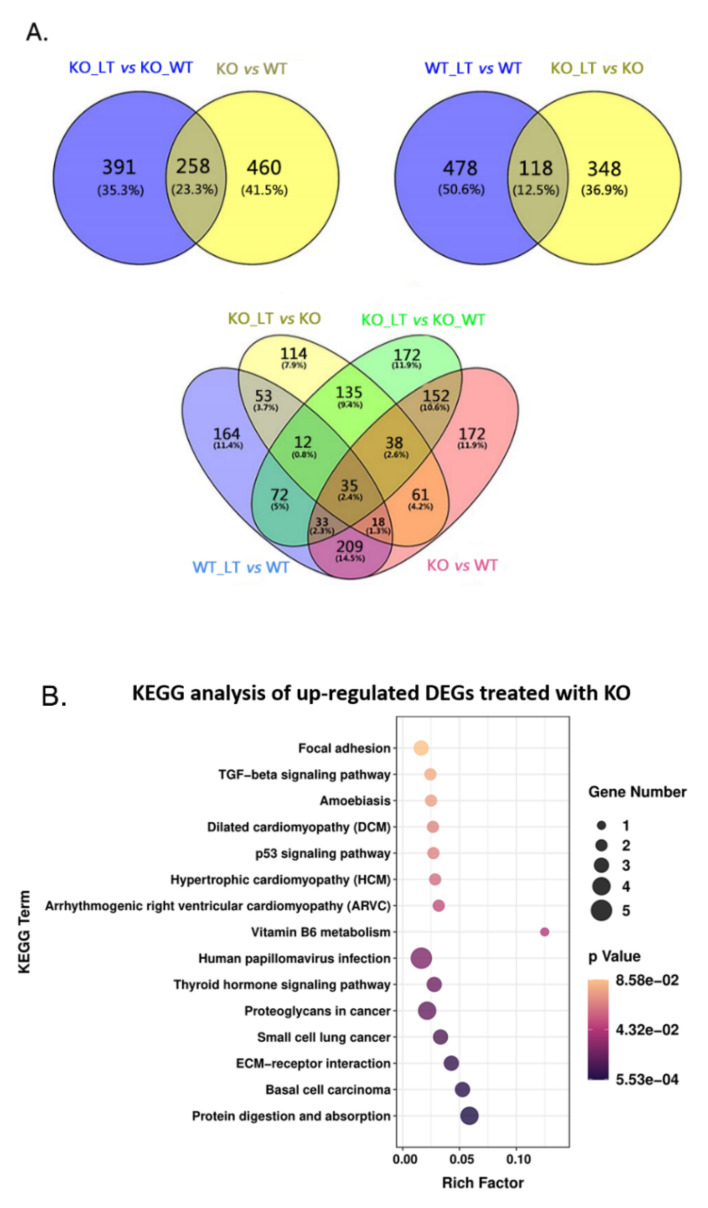

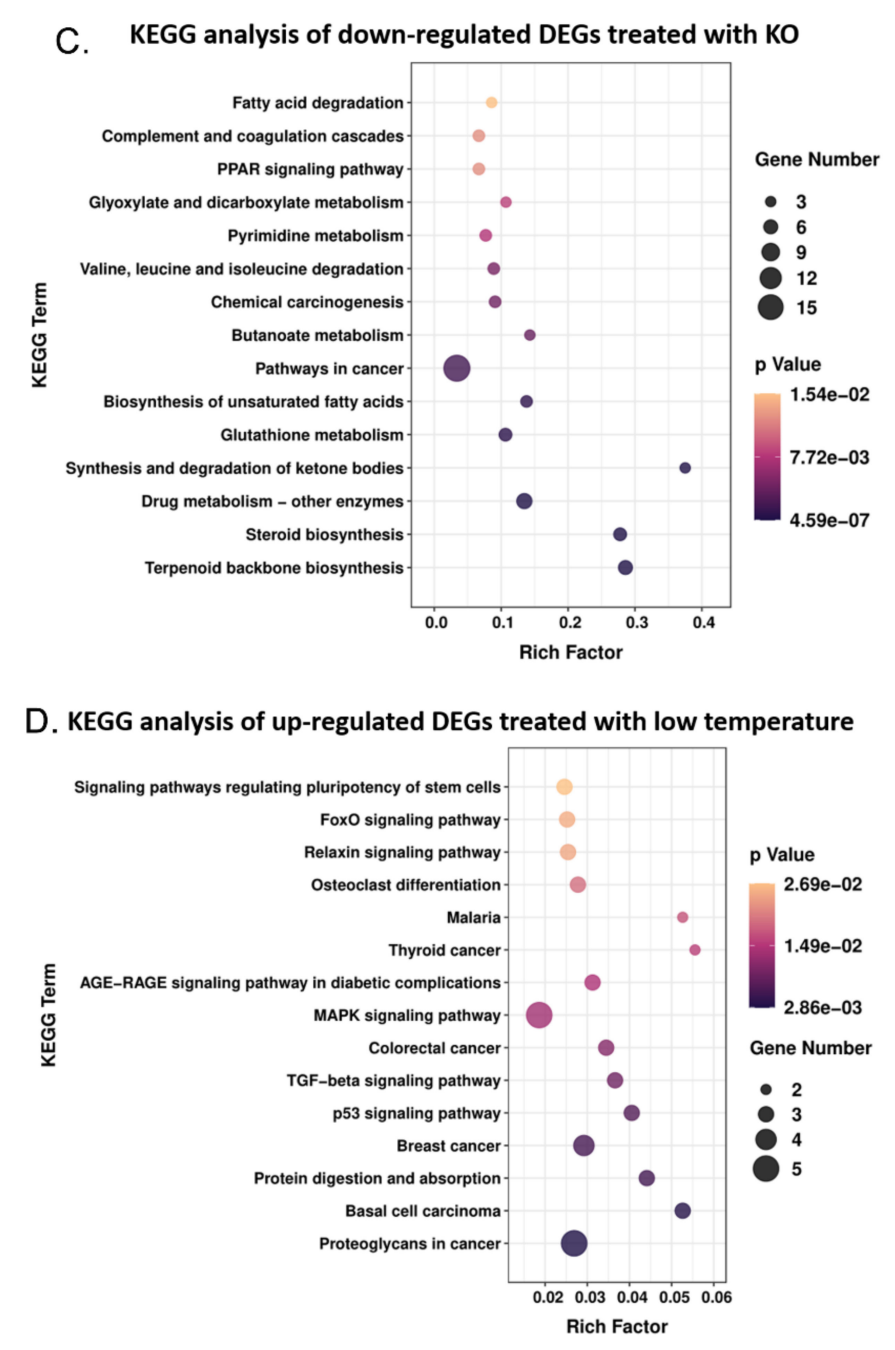

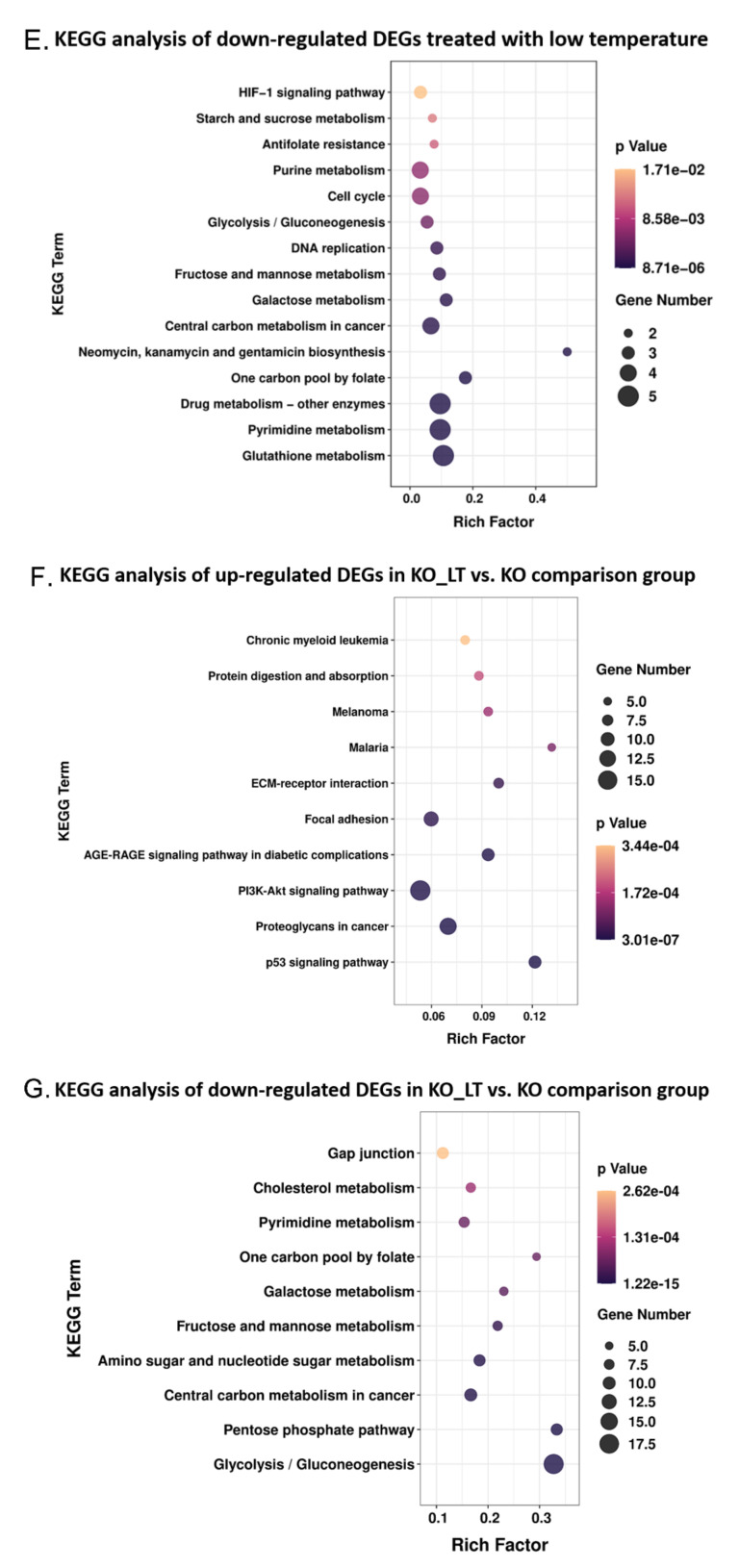

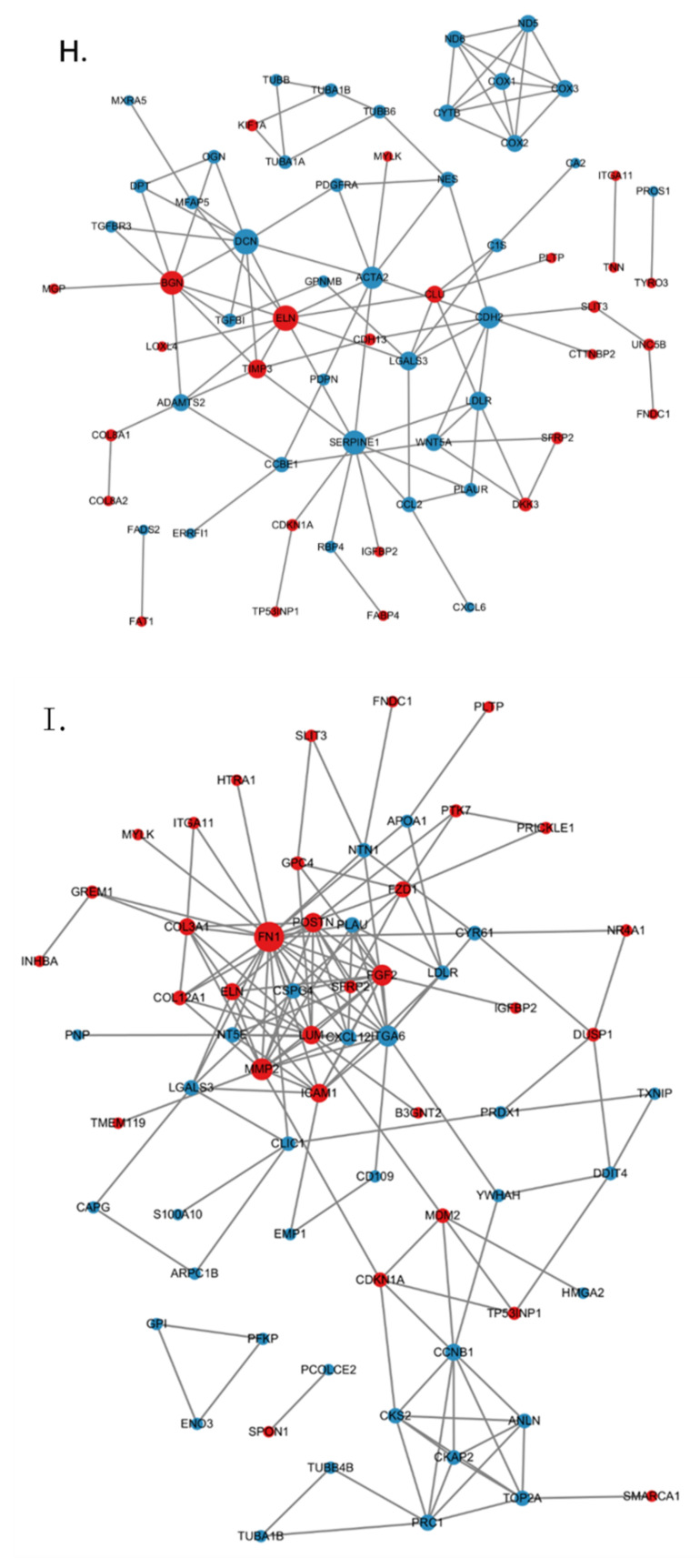
KEGG pathway analysis of DEGs in different comparisons. (**A**) Common DEGs. (**B**–**G**) Enriched KEGG pathway. (**H**) PPI network of 64 DEGs in the KO_LT versus WT_LT comparison. (**I**) PPI network of 66 DEGs in the KO_LT versus KO comparison. Red indicates up-regulated genes, and blue represents down-regulated genes.

To explore the effects of *ZC3H10* knockout on gene expression and pathways in BFFs, GO enrichment and pathway analyses were performed in the comparisons (KO vs. WT, and KO_LT vs. WT_LT; Figure 5A). KEGG analyses of up-regulated common DEGs under *ZC3H10* knockout conditions are shown in Figure 5B. The thyroid hormone signaling pathway plays an important role in growth, development, and metabolism. The thyroid hormone can alleviate cold-stress-induced apoptosis of bovine Sertoli cells through HSP70 and the mitochondrial apoptosis signaling pathway [12]. The thyroid hormone signaling pathway is closely related to thermogenesis [13]. The extracellular matrix (ECM)–receptor interaction pathway plays an important role in the morphogenesis of tissues and organs and the maintenance of the structures and functions of cells and tissues. It is also related to the process of adipogenesis [14]. The P53 signaling pathway is a part of the innate immune system and plays an important role in infectious diseases [15]. The TNF signaling pathway plays an important role in typical immune response, which is realized by regulating a series of pathways, including a direct inflammatory response with a significant innate immune participation [16]. Focal adhesion participates in numerous physiological processes and plays an important role in cell movement, cell proliferation, cell differentiation, gene expression regulation, and cell survival [17]. KEGG analysis of down-regulated common DEGs under knockout conditions are shown in Figure 5C. The synthesis and degradation pathways of ketone bodies, biosynthesis of unsaturated fatty acids, butanoate metabolism, valine, leucine, and isoleucine degradation, pyrimidine metabolism, glyoxylate and dicarboxylate metabolism, the PPAR signaling pathway, complement and coagulation cascades, and fatty acid degradation were enriched. Ketone bodies are an important energy source in non-shivering thermogenesis [18]. Unsaturated fatty acids have stimulatory effects on energy expenditure [19]. The synthesis and decomposition of unsaturated fatty acids are closely related to heat production [20]. The PPAR signaling pathway participates in the regulation of lipid metabolism, energy homeostasis, and cell differentiation, and is related to many metabolic diseases, such as metabolic syndrome, dyslipidemia, and diabetes [21].

Gene expression is a complex interaction system. To investigate whether lack of *ZC3H10* affects the interaction of genes in BFFs under low temperature, we constructed a PPI network based on 64 DEGs in the KO_LT versus WT_LT comparison by using the STRING database (https://string-db.org/, accessed on 10 March 2022) and Cytoscape (Figure 5H). This network was composed of 26 up-regulated and 38 down-regulated genes. The top four up-regulated hub genes were *ELN, BGN, TIMP3*, and *CLU*. The top four down-regulated hub genes were *DCN, SERPINE1, ACTA2,* and *CDH2*. TIMP3 is involved in thermogenesis and metabolism and influences energy expenditure [22]. In addition, six down-regulated genes (*COX1, COX2, COX3, CYTB, ND5* and *ND6*) related to the oxidative phosphorylation pathway were found. These genes are components of the mitochondrial electron transport chain (ETC) during oxidative phosphorylation, and are closely related to the production of mitochondrial energy [23]. In the ETC, along with catabolic process and effective proton gradient generation, the mitochondria provide energy for heat production [24].

### 3.6. Low Temperature-Activated Pathways Associated with Energy Metabolism and Thermogenesis Regulation

To explore the effects of low temperature on gene expression and pathway, we performed GO enrichment and pathway analyses in two comparisons (KO_LT vs. KO, and WT_LT vs. WT; Figure 5A). KEGG analysis of up-regulated common DEGs under low temperature is shown in Figure 5D. The pathway of p53 signaling pathway, TGFβ signaling pathway, MAPK signaling pathway, AGE-RAGE signaling pathway in diabetic complications, Relaxin signaling pathway, FoxO signaling pathway, and signaling pathways regulating the pluripotency of stem cells, were enriched. For example, the MAPK signaling pathway is a classical inflammatory pathway and is closely related to cell proliferation, differentiation, migration, and apoptosis [25]. Cold exposure initiates the activation of the MAPK signaling pathway and subsequently induces the up-regulation of pro-apoptotic proteins in mice [26]. The FoxO signaling pathway mediates the inhibitory action of insulin or insulin-like growth factor on key functions involved in cell metabolism, growth, differentiation, and oxidative stress [27]. FoxO and MAPK signaling pathways enhance cold resistance mainly by regulating apoptosis [28]. KEGG analysis of down-regulated common DEGs under low temperature treatments is shown in Figure 5E. The pathways of glutathione metabolism, pyrimidine metabolism, galactose metabolism, fructose and mannose metabolism, DNA replication, glycolysis/gluconeogenesis, cell cycle, purine metabolism, antifolate resistance, and starch and sucrose metabolism, and the HIF-1 signaling pathway, were enriched. Most of the DEGs were enriched in the metabolic related pathways closely related to energy metabolism and thermogenesis regulation. The HIF-1 signaling pathway is related to angiogenesis, cell proliferation or survival, and glucose or iron metabolism [29]. Evidence indicates that low temperature affects the cell cycle [30].

To explore the effect of low temperature treatment on gene expression and pathways in *ZC3H10*-deleted BFFs, we performed KEGG analysis in the KO_LT versus KO comparison. KEGG analysis of up-regulated DEGs in the KO_LT versus KO comparison is shown in Figure 5F. The p53 signaling pathway, the PI3K-Akt signaling pathway, the AGE-RAGE signaling pathway in diabetic complications, focal adhesion, and ECM-receptor interaction were enriched. The PI3K-Akt signaling pathway was activated by various types of cell stimulation. This pathway regulates the basic functions of cell. The P53 and PI3K-Akt signaling pathway can play important roles in inflammation suppression [31]. ECM-receptor interaction and focal adhesion pathway may play vital roles in cold tolerance [32]. The ECM is related to the healing of damaged tissues caused by cold stress [33]. The AGERAGE signaling pathway has complex receptors and multiple intersecting pathways, which can heavily influence cellular and systemic responses and play an important role in response to oxidative stress [34].

KEGG analysis of down-regulated DEGs in the KO_LT versus KO comparison is shown in Figure 5G. The pathways of glycolysis/gluconeogenesis, pentose phosphate pathway, amino sugar and nucleotide sugar metabolism, fructose and mannose metabolism, galactose metabolism, one carbon pool by folate, pyrimidine metabolism, cholesterol metabolism, and gap junction, were enriched. Most of these metabolism-related pathways are related to energy metabolism and thermogenesis [35].

To determine whether low temperature affects the interaction between genes in *ZC3H10* knockout BFFs, we constructed a PPI network on the basis of 66 DEGs, which was composed of 32 up-regulated and 34 down-regulated genes in the KO_LT versus KO comparison (Figure 5I). The top four up-regulated hub genes were *FN1, MMP2, FGF2* and *POSTN*. The top four down-regulated hub genes were *ITGA6, CCNB1, CXCL12* and *PRC1*. Three genes (*PLTP, LDLR,* and *FN1*) interacted with *APOA1*. *FN1* is an important gene involved in various immunity-related pathways, such as the PI3K-Akt and AGE-RAGE signaling pathways. *MMP2* plays a major role in tissue remodeling [36], and *FGF2* plays divergent roles in white adipogenic differentiation, and is a novel thermogenic regulator. The disruption of *FGF2* gene results in increased thermogenic capability in brown and beige fat [37]. *POSTN* is a component of the extracellular matrix, and loss of *POSTN* impairs lipid metabolism in adipose tissues [38].

### 3.7. Low Temperature and Deletion of ZC3H10 Altered the Gene Expression of PPAR Signaling Pathways

**Figure 6:**
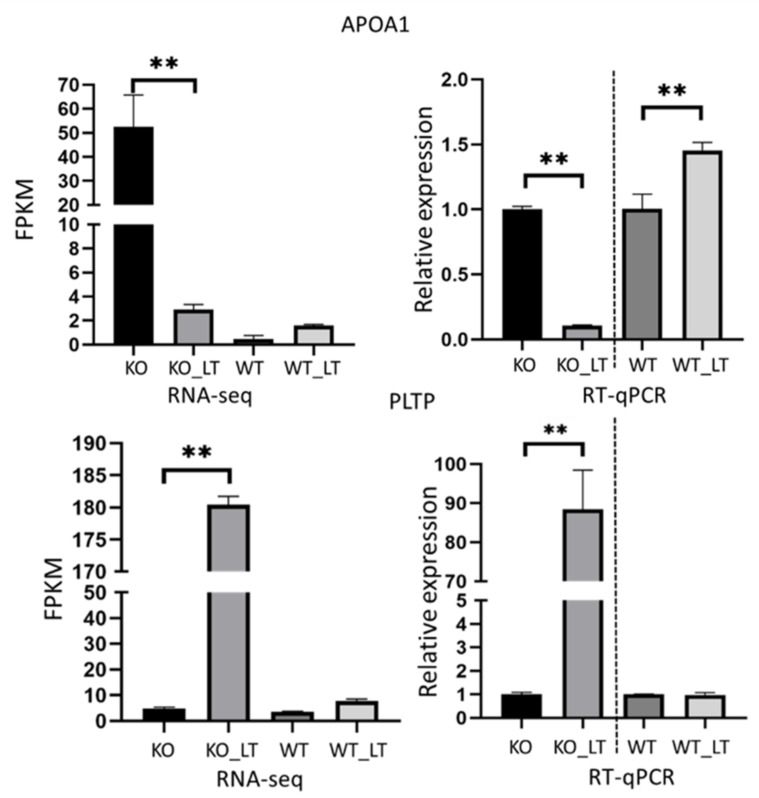
Expression levels of APOA1 and PTLP in RNA-seq and RT-PCR. ** *p* < 0.01.

In the KO_LT versus KO comparison, the expression of *LDLR* (log_2_fc = −3.52) decreased under cold stress. *LDLR* participates in lipid transport and plays a key role in energy balance [39]. The knockout of *LDLR* in mice reduces energy expenditure and promotes thermogenesis [40]. *NR4A1* (log_2_fc = 4.17) is involved in thermogenesis regulation [14]. *PLTP* (log2fc = 5.23) and *APOA1* (log2fc = −4.17) participate in the PPAR signaling pathway, which is involved in the regulation of lipid metabolism, energy homeostasis, and cell differentiation [21]. The result showed that *APOA1* expression was significantly reduced in the KO_LT group compared with the KO group (*p* < 0.01). The expression of *APOA1* significantly increased in the WT_LT group compared with the WT group (*p* < 0.01). The expression of *PLTP* significantly increased in the KO_LT group compared with the KO group (*p* < 0.01). No significant difference was found between the WT_LT and WT groups (Figure 6). The relative expression levels of *APOA1* and *PLTP* in different groups were confirmed by RT-qPCR, and the results were consistent with the RNA-seq analysis. These results suggest that loss of *ZC3H10* dsyregulated the PPAR signaling pathway under cold stress.

## 4. Discussion

In this study, we identified *ZC3H10* as a cold adaptation-related gene by selection signature analysis. We further constructed a bovine *ZC3H10*-knockout cell model and performed a comprehensive evaluation of transcriptome profile in *ZC3H10*^-/-^ fetal bovine fibroblasts under cold stress. Subsequently, we found that cold stress and loss of *ZC3H10* dysregulated the pathways and genes associated with thermogenesis and immune reaction.

The regulation of cold stress requires the joint regulation of multiple pathways. Especially, energy metabolism and immune response are two important processes involved in response to cold stress. We found that ZC3H10 was highly expressed in bovine spleen and lungs, generally consistent with previous studies [41]. The spleen is an important immune organ and plays an important role in initiating adaptive immunity [42]. Under cold stress, the gene expression of some immune-related pathways in the spleen may be changed, and the immunity of the spleen may be affected [43]. Animals need to constantly absorb oxygen and excrete carbon dioxide through metabolic processes. As a respiratory organ, the lungs play an important role in the communication between animal body and external environment. Consequently, ZC3H10 may be related to the immune response induced by cold stress. Substantial evidence indicates that cold stress affects immune regulation [44]. Long-term exposure to low temperature disrupts the body balance, and may gradually lead to internal environmental instability and increase susceptibility to diseases [45]. In the present study, we found that several DEGs were enriched in pathways related to immune regulation, such as the complement and coagulation cascade, the PI3K-Akt signaling pathway, the P53 signaling pathway, and the TNF signaling pathway. The complement and coagulation cascade pathway plays a fundamental role in innate immunity and enhances adaptive immune responses [46]. The PI3K-Akt signaling pathway is an important immune pathway, which plays a complex role in various physiological processes and pathological conditions [47]. The P53 signaling pathway is a part of the innate immune system and plays an important role in infectious diseases [15]. The TNF signaling pathway regulates typical immune responses [16]. These results imply that loss of *ZC3H10* may activate immunity-related pathways induced by cold stress. It is important to note that, through RNA-seq sequencing and differential gene comparison analysis of four combinations, we identified a number of differentially expressed consensus and specific genes; in particular, signaling pathways associated with thermogenesis and immunity; These results clarify the effects of ZC3H10 gene knockout and low temperature treatment on gene expression in cells, and also supports our hypothesis that ZC3H10 may play a role in the cold adaptation of cattle.

The cold stress response is closely related to energy metabolism. In a cold environment, cattle need to produce heat to maintain their body temperatures and increase energy consumption. The PPAR signaling pathway is one of the most important pathways involved in energy metabolism, and regulates important physiological processes, including lipid metabolism and energy homeostasis [48]. *APOA1* and *PLTP* are members of the PPAR signaling pathway and are closely related to insulin resistance, and glucose and lipid metabolism, and participate in the regulation of thermogenesis. APOA1 is mainly synthesized by the liver and intestine and plays an important role in high-density lipoprotein (HDL) biosynthesis and transport [49]. Under cold conditions, increase in energy expenditure leads to the utilization of large amounts of circulating triglycerides as substrates in ATP synthesis, and contributes to thermogenesis. In HDL transport, APOA1 affects the utilization of some substrates by changing the physiological characteristics of the cell membrane and regulating the response of membrane protein to external stimulation, has a direct influence on the energy metabolism of brown and white fat, and participates in thermoregulation by carrying some bioactive lipids [50]. PLTP is a non-specific lipid transfer protein, which can transport phospholipids, diacylglycerol, and HDL [51]. In brown fat, PLTP participates in the regulation of glucose tolerance and insulin sensitivity and is involved in heat production and glucose uptake efficiency [52]. In the present study, the results of RT-qPCR combined with RNA-seq showed that the expression levels of *PLTP* and *APOA1* changed significantly in *ZC3H10*^-/-^ fetal bovine fibroblasts. We speculated that energy metabolism-related pathways are disturbed after the knockout of *ZC3H10*. The result implies that *ZC3H10* modulates energy metabolism and lipid transport and affects adaptability to cold environments in cattle. However, the mechanisms by which *ZC3H10* functions by regulating metabolic pathways remains unclear.

## 5. Conclusions

Transcriptomic analysis highlighted the potential role of *ZC3H10* in fetal bovine fibroblasts under cold stress. This study established a gene database characteristic of the cold stress response in fetal bovine fibroblasts. The sequencing data of transcriptome can be used as a reference for further research in this field.

Our study is the first to examine low-temperature-induced changes in the transcriptome of *ZC3H10*^-/-^ fetal bovine fibroblasts, and the possible role of *ZC3H10* in bovine cold adaptation. Our findings suggest that *ZC3H10* participates in thermogenesis and immune responses under cold stress, specifically modulating immunity-related pathways and genes related to glucose and lipid metabolism and lipid transport (such as *PLTP* and *APOA1*) through metabolism-related pathways, thus generating adaptability to cold climate.

## Data Availability

Data are contained within the article and Supplementary Materials. The genotyping data presented in this study are available in Dryad deposit https://doi.org/10.5061/dryad.15dv41nvs.

## Supplementary Materials

The following supporting information can be downloaded at https://www.mdpi.com/article/10.3390/genes13101910/s1. Table S1: Information of 15 cattle breeds. Table S2: Total RNA quality inspection results. Table S3: Statistical table of transcriptome sequencing data. Table S4: Statistical table of reference genome alignment data. Table S5: DEGs were identified in four comparisons (KO_LT vs. KO, WT_LT vs. WT, KO vs. WT, and KO_LT vs. WT_LT).
