# Supplementary material for: Selection Signature and CRISPR/Cas9-Mediated Gene Knockout Analyses Reveal ZC3H10 Involved in Cold Adaptation in Chinese Native Cattle
Source: Genes (Basel). 2022 Oct 20;13(10):1910. doi: 10.3390/genes13101910 (PMC9601761; doi:10.3390/genes13101910)
Supplement: Supplementary file 1 [file genes-13-01910-s001.zip › Supplementary Tables/Supplementary Tables.pdf]

Table S1. Information of 15 cattle breeds.

Name	Abbreviation	Breeds	Sampling Location	Group	Annual Average Temperature (℃)
Yanbian	YB	taurus	Yanji, Jilin	Cold-adapted	6.24
Hazake	HSK	taurus	Qinghe, Xinjiang	Cold-adapted	1.83
Aletai White	ALT	taurus	Buerjin, Xinjiang	cold-adapted	4.99
Xinjiang Brown	XJB	taurus	Zhaosu, Xinjiang	cold-adapted	4.2
Menggu	MG	taurus	Huhehaote, Neimenggu	cold-adapted	7.6
Tibetan	TB	taurus	Lasha, Tibetan	cold-adapted (hypoxia)	9.3
Fuzhou	FZ	taurus	Wafangdian, Liaoning	cold-adapted	10.3
Anxi	AX	taurus	Jiuchuan, Gansu	cold-adapted	8.23
Apeijiaza	APJZ	taurus*indicus	Gongbujiangda, Tibetan	cold-adapted (hypoxia)	10.3
Shigatse Humped	SGH	Indicus*Taurus	Rikaze, Tibetan	cold-adapted (hypoxia)	7.23
Leiqiong	LQ	indicus	Haikou, Hainan	heat	24.73
Chuannan Mountain	CNSD	Taurus*indicus	Gongxian, Sichuan	heat	18.6
liangshan	LS	Indicus*taurus	Puge, Sichuan	heat	18.1
Ji'an	JA	indicus	Ningdou, Jiangxi	heat	20.2
Weizhou Yellow	WZ	indicus	Weizhou island, Guangxi	heat	23.6

Table S2. Total RNA quality inspection results.

Sample	OD260/280	OD260/230	Concentration（μg/μL）	Total（μg）	RIN
KO1	1.8230	1.3740	0.0413	1.7338	9.2
KO2	1.8710	1.3690	0.0540	2.2697	10
KO1_LT	1.9000	1.1540	0.0897	3.7666	10
KO2_LT	1.9310	1.5320	0.1022	4.2924	9.9
WT1	2.0690	1.9490	0.2918	12.2539	10
WT2	2.0290	1.7870	0.1761	7.3954	10
WT1_LT	2.0250	1.7660	0.1372	5.7607	10
WT2_LT	2.0380	1.8720	0.1513	6.3538	10

Table S3. Statistical table of transcriptome sequencing data.

Sample	Raw Data		Valid Data		Valid Ratio(%)	Q30（%）	GC Content（%）
	Read	Base	Read	Base			
KO1	50544962	7.58G	49492652	7.42G	97.92	98.20	50.50
KO2	55618396	8.34G	54438244	8.17G	97.88	98.19	50.50
KO1_LT	49014610	7.35G	47901990	7.19G	97.73	98.03	50.50
KO2_LT	44226758	6.63G	43258296	6.49G	97.81	98.01	50.50
WT1	50892458	7.63G	49801824	7.47G	97.86	98.11	49.50
WT2	52407024	7.86G	51282518	7.69G	97.85	98.19	49.50
WT1_LT	50922318	7.64G	49841978	7.48G	97.88	98.21	49.50
WT2_LT	52042230	7.81G	50931990	7.64G	97.87	98.21	49.50

Table S4. Statistical table of reference genome alignment data.

Sample	Valid Reads	Mapped Reads	Unique Mapped Reads	Multi Mapped Reads
KO1	49492652	48256512(97.50%)	38114603(77.01%)	10141909(20.49%)
KO2	54438244	53075602(97.50%)	41795886(76.78%)	11279716(20.72%)
KO1_LT	47901990	46535667(97.15%)	36444624(76.08%)	10091043(21.07%)
KO2_LT	43258296	42072595(97.26%)	33154778(76.64%)	8917817(20.62%)
WT1	49801824	48513425(97.41%)	38533619(77.37%)	9979806(20.04%)
WT2	51282518	50012783(97.52%)	39676661(77.37%)	10336122(20.16%)
WT1_LT	49841978	48585916(97.48%)	38403946(77.05%)	10181970(20.43%)
WT2_LT	50931990	49631851(97.45%)	38989754(76.55%)	10642097(20.89%)
